# Reinforcement, Dopamine and Rodent Models in Drug Development for ADHD

**DOI:** 10.1007/s13311-012-0132-y

**Published:** 2012-07-18

**Authors:** Gail Tripp, Jeff Wickens

**Affiliations:** 1 Human Developmental Neurobiology Unit, Okinawa Institute of Science and Technology Graduate University, Kunigami, Okinawa 904-0412 Japan; 2 Neurobiology Research Unit, Okinawa Institute of Science and Technology Graduate University, Kunigami, Okinawa 904-0412 Japan

**Keywords:** ADHD, Reinforcement, Dopamine, Rodent models

## Abstract

Attention deficit hyperactivity disorder (ADHD) presents special challenges for drug development. Current treatment with psychostimulants and nonstimulants is effective, but their mechanism of action beyond the cellular level is incompletely understood. We review evidence suggesting that altered reinforcement mechanisms are a fundamental characteristic of ADHD. We show that a deficit in the transfer of dopamine signals from established positive reinforcers to cues that predict such reinforcers may underlie these altered reinforcement mechanisms, and in turn explain key symptoms of ADHD. We argue that the neural substrates controlling the excitation and inhibition of dopamine neurons during the transfer process are a promising target for future drug development. There is a need to develop animal models and behavioral paradigms that can be used to experimentally investigate these mechanisms and their effects on sensitivity to reinforcement. More specific and selective targeting of drug development may be possible through this approach.

**Electronic supplementary material:**

The online version of this article (doi:10.1007/s13311-012-0132-y) contains supplementary material, which is available to authorized users.

## Introduction

Attention deficit hyperactivity disorder (ADHD) is a behaviorally defined disorder of unknown etiology and pathophysiology. As such, it presents special challenges to develop animal models for drug development. In this review, we consider evidence that altered mechanisms for positive reinforcement may be a fundamental characteristic of ADHD and suggest that sensitivity to positive reinforcement is a useful construct for animal models and paradigms for research into new treatments for ADHD. We have not undertaken a comprehensive review because a number of thorough reviews have recently appeared in the literature dealing separately with animal models [1–8], reinforcement theories [9–12], and psychopharmacology of ADHD [13–15]. We have instead attempted to integrate these areas by focusing on the neurobiology of positive reinforcement and its implications for new drug therapy.

For clarity in terminology, we will use the term “positive reinforcer,” or simply “reinforcer” to refer to a stimulus that increases the likelihood of an associated response, or a stimulus that would elicit an approach reaction, commonly referred to as a reward. We use the term “reinforcement learning” to refer to the associative process that occurs when a behavior is followed by a positive reinforcer. In humans, the term “primary reinforcer” is too restrictive for the reinforcing events that motivate behavior, which include social reinforcers, such as praise or money. For these events, we use the term “established positive reinforcer.” We first review behavioral aspects of ADHD and evidence for altered positive reinforcement mechanisms. We then review the neurobiology of positive reinforcement and the central role of the neurotransmitter dopamine in reinforcement learning. We consider a postulated deficit in the transfer of dopamine signals from established positive reinforcers to cues that predict positive reinforcers as an explanation for altered reinforcement sensitivity in ADHD. Next we consider rodent models for ADHD that have been evaluated in relation to reinforcement, and their potential use in drug development. This includes a review of behavioral paradigms that can be used to assess sensitivity to reinforcement. We conclude that few models have been adequately tested for altered reinforcement sensitivity, or for their ability to predict the effectiveness of new compounds for treatment of ADHD, particularly altered reinforcement sensitivity. Finally, we consider new directions for the development of animal models that could test the effects of novel therapeutic agents on dopamine responses to cues and established positive reinforcers.

## ADHD is a Behaviorally Defined Disorder

ADHD is a common and debilitating disorder diagnosed on the basis of persistent and developmentally inappropriate levels of over-activity, inattention, and impulsivity [16]. The symptoms included in the diagnostic criteria for ADHD are not specific to the disorder or abnormal in and of themselves. Inattention, impulsivity, and hyperactivity exist in the normal population, and may be normal at earlier developmental stages. Thus, producing an animal model of ADHD is not simply a matter of producing the symptoms. Any model of ADHD should also include quantitative assumptions as to what is relatively excessive. Furthermore, as abnormality is relative, the selection of appropriate controls for an animal model is as important as the selection of the model.

Rather than trying to model ADHD in a categorical way, an argument can be made for identifying component behavioral characteristics. For example, impulsivity is a component of ADHD. The ability of a drug to reduce impulsivity may predict therapeutic efficacy in ADHD, even if it does not affect other symptoms. This may be tested in a normal rat or in an animal expressing levels of impulsivity lying at the extreme of the normal range.

## Altered Reinforcement Sensitivity and ADHD

Clinical reports, together with a growing number of experimental studies, have identified differences in the manner in which children with ADHD respond to reinforcement [17]. Historically, children with ADHD have been described as less able to delay gratification and as failing to respond to discipline [18–21]. As a group, children with ADHD have been reported to perform less well under partial reinforcement schedules [22–24], and to respond more impulsively to reinforcements (i.e., to choose small immediate reinforcement in comparison with larger delayed reinforcement [25]. These early findings led several researchers to hypothesize that symptoms of ADHD arise from an altered or abnormal sensitivity to positive reinforcement (for more detail see Haenlein and Caul [18], Douglas [26], Tripp and Wickens [27], Barkley [28], Sagvolden et al. [29], and Sonuga-Barke [30, 31]). To test these hypotheses, researchers have compared the effects of reinforcement versus nonreinforcement on the cognitive task performance, psychophysiological reactivity, choice behavior, and brain activation of children, and to a lesser extent, of adults, with and without ADHD using a variety of paradigms.

Several studies report differences between children with ADHD and controls in the effects of reinforcement on cognitive task performance (for more detail see Carlson and Tamm [32], Konrad et al. [33], Luman et al. [34], McInerney and Kerns [35], and Slusarek et al. [36]). Some studies show differences in the psychophysiological responsiveness to reinforcement of children with and without ADHD [34, 37–40]. Other studies have evaluated the effects of different aspects of reinforcement (e.g., delay, frequency, and magnitude) on task performance and response allocation. Recent studies (for more detail see Sonuga-Barke et al. [10]) indicate that children with ADHD show relatively strong preferences for smaller immediate in comparison with larger delayed reinforcers, thus demonstrating greater reinforcer delay discounting [41–53]. Contextual factors, such as the length of delay, number of trials, or amount of practice may modulate these effects [48, 49, 54]. Children with ADHD perform less well than typically developing children when reinforcement is infrequent [55]. The actions of children with ADHD also appear to be more sensitive to recent instances of reinforcement [56]. Reinforcement learning in ADHD is impaired when reinforcers are not contingent, but probabilistic, and thus infrequent [57]. Learning from contingent reinforcers also seems to be impaired in children with ADHD [58].

The balance of evidence suggests that an alteration in processing of reinforcement may be a fundamental characteristic of ADHD. This characteristic has important implications for understanding the brain mechanisms underlying the disorder and for the development of effective pharmacological interventions. The neural circuitry for reinforcement learning is well-defined, with many pieces of evidence showing that dopamine is a key neurotransmitter-mediating reinforcement. Knowledge concerning reinforcement mechanisms is readily applicable to the development of animal models because reinforcement mechanisms can be studied in simpler animal organisms. Behavioral analysis of reinforcement, which has an extensive history in the context of animal learning, can be applied to measure the effects of reinforcement in humans. Thus, research demonstrating that children with ADHD differ from typically developing children in their sensitivity to reinforcement offers sound behavioral and neurobiological starting points for developing new and testing existing animal models.

## Neurobiology of Reinforcement

Considerable progress has been made toward understanding the neurobiology of reinforcement learning in recent decades. Three key findings have emerged that are of particular importance in understanding abnormal reinforcement sensitivity in ADHD. First, primary reinforcement is associated with phasic activity of dopamine neurons and a subsecond pulsatile increase in dopamine concentration, especially in the striatum. Second, such pulsatile dopamine release causes strengthening of corticostriatal synapses, the degree of which is correlated with the rate of learning. Third, this strengthening effect of dopamine has precise timing requirements, such that delays in the dopamine pulse result in the loss of strengthening effect. We summarize the evidence for these points in the following. This complex and emerging literature has been recently reviewed [59–63].

Animal studies have identified dopaminergic circuits in the midbrain, particularly the nigrostrial and mesolimbic projections, as being critical in reinforcement mechanisms [63, 64]. Dopamine neurons demonstrate activation in response to primary reinforcers. The phasic firing is caused by events of motivational significance, such as unexpected primary reinforcers and stimuli that predict reinforcers during learning [65, 66]. Although dopamine neurons are sometimes activated by aversive stimuli, the majority of dopamine neurons are inhibited by these stimuli [67]. Consistent with the findings in nonhuman primates, rat dopamine cells have also been shown to respond to appetitive stimuli [68]. Dopamine release in response to visual and olfactory stimuli associated with natural reinforcers has been demonstrated in the rat nucleus accumbens [69–71] and striatum [72]. However, dopamine cell firing activity does not simply follow the delivery of positive reinforcers. Established reinforcers initially evoke dopamine cell firing. With repeated repetition and learning of a task, the dopamine burst that initially occurs at the time of reinforcement transfers to earlier and earlier predictors of reinforcement [65]. In this manner, dopamine cells fire in anticipation of reinforcers (i.e, in response to cues that predict the delivery of reinforcement).

At the cellular level, the phasic dopamine release that occurs in response to reinforcement is thought to lead to strengthening of connections at the synaptic level. Several authors have suggested that dopamine-dependent synaptic plasticity mechanisms in the neostriatum may underlie such strengthening [73–77]. In support of this, experimental studies have shown that a conjunction of cortical presynaptic and striatal postsynaptic activity combined with pulsatile application of dopamine, such as that caused by unexpected primary reinforcement, leads to long-term potentiation (LTP) of corticostriatal synapses [78, 79]. Similarly, stimulation of the substantia nigra pars compacta *in vivo* induces LTP [80, 81]. Consistent with these findings, dopamine depletion or dopamine receptor antagonists can block LTP [82, 83]. For example, LTP is reduced where there is decreased synaptic release of dopamine, and is restored by dopamine receptor stimulation [84]. These findings together show that dopamine may act at a cellular level to strengthen corticostriatal synapses.

The timing of dopamine release is critical for this dopamine-mediated strengthening of synapses. Direct measures of the timing requirements for potentiation have shown that dopamine pulses must occur within subsecond intervals of the synaptic activity that is being potentiated [79]. Similar temporal requirements have been shown using dopaminergic reinforcement of single cell activity in the hippocampus [85]. Recently, studies of spike-timing dependent plasticity have been conducted in the corticostriatal pathway. These have produced interesting but conflicting results. Fino et al. [86] measured spike-timing dependent plasticity of corticostriatal connections in rat brain slices. They reported that LTP was induced when a striatal postsynaptic action potential preceded stimulation of corticostriatal, presynaptic inputs. Conversely, LTD was induced when the postsynaptic action potential came after the cortical stimulation. In contrast, Pawlak and Kerr [87] found that temporal requirements are almost the reverse of those reported by Fino et al. [86]. This is an area of ongoing research that is presently unresolved.

In summary, at the cellular level, dopamine acts by modulating or controlling synaptic plasticity. Phasic increases in dopamine concentration, which occur with reinforcer-related release of dopamine, in association with synaptic activity cause LTP of corticostriatal synapses [60, 78, 88]. However, this potentiating effect has strict temporal requirements for the dopamine pulse and synaptic activity. Taken together, these findings define a cellular mechanism for the effects of reinforcement in which phasic and appropriately timed pulses of dopamine strengthen synaptic connections.

At the behavioral level, a delay between the response or stimulus and the reinforcer results in less rapid learning [89–91]. However, learning can be enhanced under conditions of delayed reinforcement by a distinctive environmental cue, which reliably precedes delivery of the final reinforcer. The transfer of dopamine responses to the cue may be a mechanism to compensate for delay of reinforcement. When predictive cue signals are not available, the timing requirements for behavioral reinforcement are determined by the timing requirements for cellular reinforcement. For example, when electrical stimulation of the brain is used as the reinforcer delays of as little as 1 second may impair the effect of reinforcement in rats [92].

In situations in which reinforcement is delayed, the anticipatory dopamine release is thought to bridge the delay between cues and reinforcement, ensuring that the dopamine pulses occur with the required timing at the cellular level [27, 93]. We propose that in normal humans, as in nonhuman primates and rats, dopamine cell responses transfer from established reinforcers to earlier cues in the behavioral sequence, and this is important for learning with delay of reinforcement.

## Dopamine Transfer Deficit, a Neurobiological Theory of Altered Reinforcement Sensitivity in ADHD

Reward dysfunction has been proposed in a number of different theories of ADHD, including the Cognitive-Energetic model [94], the Dual Process theory [30, 31], and the Dynamic Development Theory [95]. These are general theories of ADHD that acknowledge altered reward sensitivity as an important component. More specific computational theories have also been proposed, drawing on recent models of reinforcement learning (for more detail see Luman et al. [9]).

Drawing on the extensive evidence linking dopamine cell activity to positive reinforcement, we formulated the dopamine transfer deficit (DTD) hypothesis as an explanation for altered processing of positive reinforcement in children with ADHD [27, 93]. The background of this theory is the neurobiology of reinforcement previously described. We assume that in normal humans, as in nonhuman primates and rats, the same 3 processes occur: 1) phasic dopamine signals that transfer from established positive reinforcers to earlier cues that predict reinforcers, 2) dopamine-dependent strengthening of connections, and 3) a precise timing requirement for the dopamine signal. Thus, the repeated experience of a cue followed by a reinforcer leads to transfer of dopamine cell responses from established reinforcers (e.g., praise or attention to earlier cues in the behavioral sequence that predict the later delivery of reinforcement). When this process occurs normally, it provides a mechanism of ensuring that the timing of the dopamine signal at the cellular level is immediate and continuous even when behavioral reinforcement is delayed or discontinuous. This immediate and continuous dopamine signal ensures that even in a natural environment in which reinforcers fluctuate according to the circumstances, the cellular mechanism is able to engage effectively and maintain reinforced behavior.

In ADHD, we propose the transfer of the dopamine response to previously neutral cues is disrupted. In particular, we assume that the phasic dopamine cell response to cues that predict reinforcement is reduced in amplitude to the point of being ineffective (i.e., there is a failure of the mechanism that normally comes to excite the dopamine cells in response to cues that repeatedly and persistently precede reinforcement). In addition, we assume there is a failure of the mechanism that inhibits dopamine cell firing at the time of the primary reinforcement. The net result of these 2 assumptions is a failure of the dopamine signal to transfer to the earlier cues that predict reinforcement. Instead, the dopamine signal continues to occur at the time of established positive reinforcers. Moreover, if the reinforcer is intermittent or delayed, the dopamine signal is likewise intermittent and delayed. We argue as follows that this may cause key symptoms of ADHD.

The exact nature and cause of the failure is currently unknown. However, there are many ways that such failure might occur. For example, altered dopamine transporter function, or altered dopamine receptor function, as a consequence of genetic polymorphisms in these molecular mechanisms, could disrupt the transfer process. Other possibilities also exist, such as abnormalities in the systems that are afferent to the dopamine cells, which may include prefrontal cortical mechanisms. The precise cause of the deficient transfer, however, is a separate matter from the symptoms that can be predicted from it.

We predict that such failure would lead to a number of symptoms of ADHD. As a consequence of failure to predict reinforcers, individuals with ADHD experience a delayed dopamine signal (delayed reinforcement), or no signal (discontinuous reinforcement) at the cellular level, rather than the immediate anticipatory dopamine signal experienced by typically developing individuals. This would make them abnormally sensitive to delayed or discontinuous reinforcement. It would also have the effect of making individual instances of actual reinforcement more salient, which may result in greater control by incidental stimuli in the environment over the integrated history of reinforcement. These corollaries are developed more fully in earlier theoretical articles [27, 93, 96]. DTD is only one of several recent theories addressing the neurobiology of altered reinforcement sensitivity and ADHD, which have also been reviewed in detail by Luman et al. [9]. This is emphasized here because its predictions are testable and have implications for the selection of animal models for developing new drug treatments for ADHD.

The assumption that dopamine signaling in humans parallels that measured in experimental animals has some support from functional magnetic resonance imaging (fMRI) studies. Changes in blood flow and oxygenation measured by fMRI signals are thought to provide an indirect measure of dopamine release. In particular, dopamine may activate postsynaptic neurons by potentiation of corticostriatal synapses, and therefore increase local fMRI signals [97]. Functional activations in the dorsal striatum occur in relation to reinforcement of an action [98, 99], and activation of the nucleus accumbens in relation to anticipation of reinforcement [100].

Recent functional studies in humans have been enhanced by the use of models based on the temporal difference algorithm for predicting reinforcement. This machine-learning algorithm generates prediction error signals that correlate with dopamine signals in primates during Pavlovian conditioning [101]. In human studies, responses to cues predicting a juice reinforcer have been measured in the ventral and dorsal striatum. The ventral striatal area was affected by prediction error (estimated from a temporal difference model) in both Pavlovian and instrumental conditioning, whereas the dorsal striatal was affected by instrumental conditioning [102, 103].

Evidence from recent fMRI studies provides support for the DTD hypothesis of impaired anticipatory dopamine cell firing in ADHD. In normal subjects, preference for immediate in comparison with delayed reinforcement is associated with the magnitude of ventral striatal activity [104]. Adolescents [105] and adults [106–108] with ADHD show reduced activation in the ventral striatum in response to cues predicting reinforcement in monetary incentive delay tasks [105, 106, 108] and in a temporal discounting task [107]. Stoy et al. [109] also reported a larger neural response in the putamen of adult control participants *versus* those with ADHD during gain anticipation with a monetary incentive delay task. These studies are consistent with the assumption of reduced response to cues.

Evidence for the assumption that dopamine cells continue to fire in response to established positive reinforcers is more equivocal. Strohle et al. [106] observed increased activations of the orbitofrontal cortex in ADHD participants in response to reinforced outcome. Neither the studies of Scheres et al. [105] nor Stoy et al. [109] found group differences in response to reinforcement, however, their studies were not designed to test DTD. In a study designed to test DTD assumptions directly, Furukawa et al. [110] reported preliminary findings that are consistent with impaired transfer of responses from reinforcers to cues predicting reinforcement.

There are several ADHD symptoms that can be interpreted as arising from a dysfunction in the transfer of dopamine cell firing. Symptoms of inattention include: “often fails to give close attention to details or makes careless mistakes in schoolwork, work, or other activities”; “often has difficulty sustaining attention in tasks or play activities” and “is often easily distracted by extraneous stimuli.” These symptoms occur with higher frequency in children with ADHD, and may be explained by impaired anticipatory dopamine release. The reason for this is that if the dopamine release occurs only in response to actual instances of reinforcement, incidental aspects of the environment that activate dopamine neurons may reinforce off-task behaviors rather than the socially established cues present in classroom situations. Other symptoms of inattention can be interpreted as failure of conditioned reinforcers leading to increased sensitivity to delay of reinforcement and less effective performance under schedules of partial reinforcement. These include: “often does not follow through on instructions and fails to finish schoolwork, chores, or duties in the workplace,” and “often avoids, dislikes, or is reluctant to engage in tasks that require sustained mental effort.”

The DTD theory also explains some symptoms of hyperactivity and impulsivity. The symptom of “often leaves seat in classroom or in other situations in which remaining seated is expected” may be interpreted as a lack of effective reinforcement for remaining seated arising from a deficit in anticipation of reinforcement. The symptoms “often has difficulty awaiting turn,” “often blurts out answers before questions have been completed,” and “often interrupts or intrudes on others” involve a delay between the target behavior and the actual reinforcement. Therefore, they may be interpreted as being due to abnormal sensitivity to delay of reinforcement arising from a failure of the transfer of dopamine cell firing activity in response to cues that predict reinforcement.

Although many ADHD symptoms can be explained by DTD, it would be possible to have DTD and not meet criteria for ADHD, because some symptoms might arise by different mechanisms. However, DTD does provide a distinct framework for the development of animal models and new targets for pharmacological compounds for a particular set of symptoms.

## Current Drug Treatment of ADHD

Because the neurotransmitter dopamine is involved in the processing of reinforcement and reinforcement learning, it is an important system in the development of new medications to treat ADHD. In particular, the importance of timing requirements suggests that ADHD medications should target anticipatory release of dopamine. However, the primary action of the drugs currently used in the treatment of ADHD is an action on dopamine signals, which is not specific to the timing or cause of the dopamine signal. They may compensate for a deficit in dopamine transfer, but do not correct the primary deficit of failure to transfer. Such specificity of action may not be possible with drugs that act on the dopamine transporter rather than the circuitry that controls the dopamine neurons.

The molecular actions of existing drugs used in the management of ADHD provide limited insight into the underlying pathology of ADHD. It is commonly assumed that because these drugs are indirect dopamine agonists, there must be an overall deficiency of dopamine. However, no such overall deficiency has been conclusively identified, and more subtle alterations in dopamine function, such as DTD may be important for the development of new variants of these drugs and novel therapeutic compounds. Currently 2 major classes of drugs are used in the treatment of ADHD: 1) psychostimulants and 2) norepinephrine reuptake inhibitors. Amphetamine (Adderall) and methylphenidate (Ritalin, Concerta) are psychostimulants. Atomoxetine (Strattera) is a norepinephrine reuptake inhibitor.

At therapeutic dosages, an amphetamine is a dopamine reuptake inhibitor and also a norepinephrine reuptake inhibitor, and it is also a very weak inhibitor of serotonin reuptake [111]. At higher doses, an amphetamine also evokes the release of dopamine, norepinephrine, and 5-hydroxytryptamine. Similarly, methylphenidate (dl-threo-methylphenidate) is a reuptake inhibitor for dopamine and norepinephrine, while it is inactive for 5-hydroxytryptamine. Both drugs are very effective in reducing the symptoms of ADHD [112–114], but the therapeutic effects require the presence of the drug and do not outlast drug exposure [13].

Atomoxetine (Strattera) is a nonstimulant that acts as a presynaptic blocker of norepinephrine reuptake [115], increasing the duration of action of norepinephrine after its release. Atomoxetine also increases dopamine concentration because the norepinephrine transporter plays an important role in clearance of dopamine in this region [115, 116], but this is not so in the striatum, and therefore, the effects on dopamine are regionally selective. Thus, atomoxetine increases dopamine, as well as norepinephrine in the prefrontal cortex, but not in the striatum where the dopamine transporter plays the major role in clearance.

Volkow et al. [117] showed that a standard clinical dose of 0.5 mg/kg methylphenidate blocks approximately 60 % or more of the dopamine transporter. Other studies also suggest that clinically relevant doses of methylphenidate produce their therapeutic effects by increasing extracellular dopamine [118–120]. Volkow et al. [121] proposed that the therapeutic effects of methylphenidate may be “secondary to its ability to enhance stimuli-induced dopamine increases, thus making them more motivationally salient and thereby improving performance.”

Human studies support suggestions that actions on reinforcement mechanisms underlie the therapeutic effects of methylphenidate. In children with ADHD, methylphenidate has been shown to raise the breakpoint (i.e., the point at which the participant elects to stop responding) on progressive ratio tasks [122, 123]. Methylphenidate has also been shown to reduce delay discounting (i.e., preference for small immediate delayed reinforcers in comparison with larger delayed reinforcers in children with ADHD during a real-time discounting task [124].

## Rodent Models for ADHD

A number of different rodent models have been proposed, and there are several comprehensive reviews [1–8, 125, 126]. Here we focus on those most relevant to reinforcement theories. The main approaches include: 1) developing measures of the altered reinforcement responses in ADHD and studying the effects of drugs on these measures in normal animals; 2) selecting for behavioral characteristics by selective breeding or selection of phenotypes from the natural variation in sensitivity to reinforcement; 3) mimicking presumed pathology by making brain lesions of brain reinforcement circuitry; and 4) manipulation of candidate genes and the production of transgenic animals. These approaches are considered as follows.

### Behavioral Paradigms Used to Model ADHD and Drug Actions

The symptoms of ADHD are not unique to it and are present in some degree in the normal population. Similarly, psychostimulant drugs modify attention, impulsivity, and hyperactivity in people with and without ADHD. The use of “normal” rats, with appropriate behavioral measures, is thus well justified. A number of excellent reviews of behavioral paradigms are available (for more detail see Evenden [127, 128] and Winstanley et al. [129]). As follows, we describe the most frequently used paradigms and the effects of existing drugs on these measures.

#### Delay Discounting

The delay-discounting paradigm measures the tendency to choose an earlier, smaller reinforcer in preference to a larger, later reinforcer. In general, organisms discount future reinforcers as a function of the delay from the time of choice [130], choosing immediate reinforcers, even if this results in fewer total reinforcers [127]. A number of studies have investigated the effects of psychostimulants on the performance of normal rats in delay-discounting tasks.

Bizot et al*.* [131] trained rats in a T-maze to choose between a smaller immediate reinforcer and a larger reinforcer that was delayed by 30 seconds. Methylphenidate (3 mg/kg), atomoxetine (1 mg/kg), d-amphetamine (1 and 2 mg/kg), and desipramine (8 and 16 mg/kg) all led to an increased choice of the larger delayed reinforcer. The authors suggest that the T-maze procedure in juvenile animals may be suitable for testing the therapeutic potential of drugs for the management of ADHD [131].

#### 5-Choice Serial Reaction Time Test

The 5-choice serial reaction time test (5-CSRT) is a rat version of the continuous performance test used in humans [132]. In this task, the animal is required to learn to nose-poke into 1 of 5 apertures after the presentation of a brief visual stimulus in that aperture to obtain a food reinforcer. The stimulus is short in duration, requiring the rat to “attend” closely. Premature responses reflect higher levels of impulsivity and provide a measure of behavioral inhibition.

The 5-CSRT can be used to identify rats within a “normal” population with a deficit in selective attention accompanied by impulsivity. Rats selected for high levels of impulsivity on a 5-CSRT task also exhibited high levels of impulsive decision-making on a delay-of-reinforcement task [133]. However, the same rats were not impaired on a stop-signal task requiring inhibition of an already initiated motor response [134]. These animals have been proposed as a rodent model of ADHD.

Puumala et al. [135] used a 5-CSRT to assess sustained attention, measured by choice accuracy, and motor hyperactivity, measured by percentage of premature responses. At lower doses, methylphenidate slightly improved sustained attention in poorly performing animals, but at higher doses (1 mg/kg) it increased the number of premature responses. Similarly, Navarra et al. [136] found that methylphenidate at therapeutic doses improved sustained attention. Cole and Robbins [137, 138] reported that methylphenidate at higher doses increased premature responding on the 5-CSRT, whereas atomoxetine induced a marked decrease in impulsivity and overall improvement in attention. The different effects of methylphenidate and atomoxetine may reflect different involvement of prefrontal and striatal regions in these tasks.

#### Stop Signal Reaction Time Task

Stop-signal reaction time tasks (SSRTs) measure the ability to withhold or inhibit an already initiated or pre-potent motor response [139]. Stop-signal tasks are similar to “go/no go” tasks, in which the stop signal is presented before or simultaneously with the go signal. Behavioral paradigms for both SSRT and “go/no go” have been developed for use in animal studies [140, 141]. The effects of psychostimulants on these paradigms were recently reviewed [142]. Psychostimulants and atomoxetine decrease impulsivity on the SSRT [143], suggesting that this task may be useful in animal studies of potential therapeutic agents.

### Rodent Models Developed through Selective Breeding

The spontaneously hypertensive rat (SHR) is the most widely used animal model of ADHD [144–149]. SHRs are more impulsive than their control strain, Wistar Kyoto (WKY). Bizot et al. [150] found that adult SHR are more impulsive than WKY or Wistars in a T-maze, in which rats had to choose between a small, but immediate and a large, but delayed reinforcer. Fox et al. [151] used a similar procedure and found that the SHRs chose more small/immediate reinforcers than the WKYs at the longest delays. Similarly, Pardey et al. [152] found that SHRs were more sensitive to delay of reinforcement than WKYs on an operant task, allowing free choice between a small reinforcer delivered immediately and a larger reinforcer delivered after a delay.

Closely related to the delay-discounting paradigm, Sutherland et al. [153] adapted a signal detection task used to measure the effects of delay of reinforcement in children [56] for use with rats. In the task, the rat is required to press 1 of 2 available levers on each trial. One lever delivers an immediate reinforcement, whereas the other lever delivers a reinforcement after a fixed delay. Sutherland et al. [153] found that the SHRs were more sensitive to delay of reinforcement than control strains. These findings collectively support the SHR as a model for impulsive behavior. However, these comparisons were made against various control strains, the normality of which can be difficult to establish.

There have been relatively few studies of the effects of psychostimulants on the SHR. In the T-maze task, previously described, in which rats had to choose between a small, but immediate and a large, but delayed reinforcer procedure [150], the SHR exhibits more impulsive behavior. Bizot et al*.* [150] found that methylphenidate 3 mg/kg did not reduce impulsivity in the SHR. Kantak et al*.* [154] investigated the effects of oral methylphenidate (1.5 mg/kg) on delayed win-shift (nonspatial working and reference memory), win-stay (habit learning), and attentional set-shifting (attention and behavioral flexibility) tasks. On all 3 tasks, the SHR made significantly more errors than the WKY. Treating the SHR with methylphenidate eliminated strain differences in all 3 tasks. Liu et al. [155] investigated the effects of atomoxetine on the behavior of the SHR in the Morris water maze. Maze learning was improved after atomoxetine administration. The performance of the SHR has also been tested in the 5 choice serial reaction time paradigm, but this was not abnormal. Methylphenidate (0.1-1.0 mg/kg) did not improve performance of SHR in this task [156].

Similar to the SHR, a genetically independent, hypertensive rat strain, known as the Genetically Hypertensive (GH) rat, was developed in New Zealand by selective breeding of Wistar rats for hypertension [157–159]. The GH showed no evidence of hyperactivity within an open field in comparison to its parent strain, the Wistar [160–162]. Wickens et al. [163] tested the GH strain using the FI-EXT task that has been used extensively in studies with the SHR. As with the SHR, the GH showed higher terminal response rates and response bursts, and a greater level of continued responding during EXT, in comparison to both the Wistar and WKY strains. In a direct measure of sensitivity to delay of reinforcement, Sutherland et al. [153] found that both SHR and GH rats allocated significantly more responses to the immediately reinforced lever than their genetic control strains. These findings support the use of both SHR and GH rat to model altered reinforcement in ADHD.

The GH provides an interesting complement to the SHR, in that in both strains the hyperactivity has arisen from the selection for high blood pressure, although is not related to blood pressure per se. This convergence across strains suggests that the relevant genes may be physically close, but they are not identical to those for hypertension. However, further work is needed to analyze the behavioral characteristics of the GH rat.

### Selective Lesion Rodent Models

Neurochemically, selective lesions of the dopamine neurons by neonatal administration of the neurotoxin 6-hydroxydopamine (6-OHDA) lead to locomotor hyperactivity [3, 164]. The hyperactivity induced by neonatal 6-OHDA lesions is reduced by amphetamine [165] and methylphenidate [166, 167]. Kuczenski and Segal [168] established psychostimulant doses and conditions that approximated clinically relevant conditions. They found that low, oral doses of methylphenidate, which produce blood levels similar to those in patients with ADHD, decrease locomotor activity in juvenile rats [168, 169]. These low doses have a preferential effect on the norepinephrine transporter, increasing norepinephrine levels in the prefrontal cortex [170]. Altered norepinephrine in the prefrontal cortex, may in turn modulate dopamine release in response to reinforcer predicting cues.

A 6-OHDA mouse model has also been developed, which shows increased activity levels and a reduction in activity after treatment with psychostimulants [171, 172]. The neonatal 6-OHDA lesioned animal model has not yet been validated using tasks that measure behavioral characteristics, such as impulsivity or sensitivity to delay of reinforcement.

### Transgenic Animal Models

Several pieces of evidence suggest that abnormal dopamine transporter (DAT) function may be important in ADHD. Overexpression of DAT has been found in human ADHD, and 1 of the major actions of the psychostimulants is to block DAT. Thus, DAT dysfunction has been proposed as a possible cause of ADHD, and mice with genetically engineered changes of DAT function have been proposed as models for use in ADHD research [173–175]. For example, DAT knockout mice show increased locomotion. In the open-field environment, high doses of methylphenidate (30 mg/kg) increased locomotion in wild-type mice and decreased locomotion in DAT knockout mice. The effects in knockout mice were mediated by increases in serotonin, suggesting that therapies directed at serotonin may be useful in ADHD [176]. Although genetic engineering offers powerful approaches to develop models, only a small fraction of variance in human populations is accounted for by variation in dopamine transporter, and unconditionally increased locomotor activity is not a key component of ADHD.

## Future Directions

Based on the increasing evidence that altered reinforcement mechanisms may underlie or be associated with some symptoms in ADHD [27], we suggest that future efforts to develop drug treatments may be usefully focused on these mechanisms. Compounds of potential value can be identified by their effect on animal tasks that measure reinforcement variables. Strategies may include further characterization of the altered reinforcement processing in children with ADHD and the translation of tasks into reliable and valid animal behavior paradigms.

The DTD hypothesis identifies certain neural targets for intervention in ADHD. In particular, the circuitry mediating excitation of dopamine neurons by cues predicting reinforcement is a potential target. Candidate structures include the basolateral amygdala projection to the nucleus accumbens, [177] central nucleus of the amygdala projection to the dorsolateral striatum, and [178] pallidal projection to the lateral habenular [179–181], in addition to more commonly understood projections from the prefrontal cortex and pedunculopontine nucleus. Synaptic plasticity in this circuitry may mediate the association of cues with dopamine release. Understanding the mechanisms of synaptic plasticity in this circuit may be important for development of new drugs. At the same time, the circuitry mediating inhibition of the dopamine neurons at the time of primary reinforcement is another strong candidate target of therapy. Although it may be difficult to elucidate these mechanisms in the short term, it should be possible to test whether disrupting these circuits leads to altered sensitivity to reinforcement and behavioral characteristics of ADHD. This requires the use of tests of sensitivity to reinforcement that have been translated from human to animal paradigms. Another possibility would be to investigate differential activation of circuits in animals that exhibit extremes of sensitivity to reinforcement. Compounds that correct activity in these circuits or produce compensatory changes in other circuits are candidates for further development.

The identification of the neurobiological substrates underlying reinforcement by basic research suggests more specific and selective targeting of drug development may be possible, focusing on the mechanisms that mediate anticipation of reinforcement. The therapeutic effects of existing drugs can be understood in this framework, but there are likely to be improvements within their wide spectrum of action on neuromodulators. For example, blocking dopamine transporter activity probably increases dopamine signals evoked by both cues and established positive reinforcers. According to the DTD hypothesis, a better result would be to increase the dopamine signal in response to the cues, and decrease the signal in response to the established positive reinforcer. To achieve this it would be necessary to target the neural circuitry that excites the dopamine neurons in response to the cues (and enhance its effects), and separately target the circuitry that inhibits the dopamine neurons at the time of the reinforcer. To date, we know of no treatment designed to produce these effects.

Advances in understanding the pathophysiology of ADHD in terms of altered reinforcement sensitivity may also lead to the development of animal models of specific components of the ADHD syndrome. This may range from the selection of phenotypes from normal populations, selective breeding, genetic manipulation, or specific lesions. Another possibility is optogenetic inhibition of dopamine cell firing at the time of cue presentation, directly simulating DTD.

With these advances, it may be possible to target specific symptoms of ADHD for selective treatment. It is well known that ADHD is a heterogeneous disorder that overlaps with other behavioral disorders. Treatments that target reinforcement mechanisms are unlikely to cover all aspects of the disorder. However, such treatments may reduce specific symptoms or processes that affect learning and socialization.

## Electronic Supplementary Material

Below is the link to the electronic supplementary material.

Disclosure forms (PDF 510 kb)
