# Supplementary material for: Reinforcement, Dopamine and Rodent Models in Drug Development for ADHD
Source: Neurotherapeutics. 2012 Jul 18;9(3):622–34. doi: 10.1007/s13311-012-0132-y (PMC3441939; doi:10.1007/s13311-012-0132-y)
Supplement: Supplementary file 1 — (PDF 510 kb) [file 13311_2012_132_MOESM1_ESM.pdf]

To view the full contents of this document, you need a later version of the PDF viewer. You can upgrade to the latest version of Adobe Reader from www.adobe.com/products/acrobat/readstep2.html

For further support, go to www.adobe.com/support/products/acrreader.html
